# Supplementary material for: Empirical methods for controlling false positives and estimating confidence in ChIP-Seq peaks
Source: BMC Bioinformatics. 2008 Dec 5;9:523. doi: 10.1186/1471-2105-9-523 (PMC2628906; doi:10.1186/1471-2105-9-523)
Supplement: Additional File 1 — A variety of html documents from the USeq web site detailing the available applications, their best usage, output file type descriptions, command line menus, etc. [file 1471-2105-9-523-S1.zip › USeqUserGuides/JavaDoc.zip!/JavaDoc/trans/misc/package-frame.html]

trans.misc


trans.misc

Classes 
  
AverageDoubles
  
Bar2Gr
  
Bed2Gr
  
BestWindowScoreExtractor
  
CalcGCAndTransform
  
CalcGCRatiosFromIntervals
  
CalculateGeneExtensions
  
ConvertAgilentData
  
ConvertBars2TPMapCel
  
ConvertGeoData
  
ConvertNimblegenNDF2TPMap
  
ConvertNimblegenPAIR2Cela
  
CorrelateGrFiles
  
Delme
  
GCRatio
  
Gr2Bar
  
GrGraph
  
InefficientPseudoMedian
  
PrependColumn
  
Regions2Intervals
  
ScoredRegionsFormatFixer
  
Sgr2Bar
  
SgrComparator
  
TextPValOrSig2Gr
  
TransformRatios
  
Util
  
Wig2Bar
  
WindowHistogram
